# 1051. Characterization of Immune Responses to a Live-Attenuated Tetravalent Dengue Vaccine

**DOI:** 10.1093/ofid/ofab466.1245

**Published:** 2021-12-04

**Authors:** Ralph Braun, Mayuri Sharma, Christina DeMaso, Allan Parker, David Dominguez, Heather Watkins, Hansi Dean, Lovkesh Karwal, Eduardo Nascimento, Nicole Messere, Isamu Tsuji, Melissa Zahralban-Steele, Jeffrey R Currier, Heather Friberg-Robertson

**Affiliations:** 1 Takeda Pharmaceuticals, Stow, Massachusetts; 2 Walter Reed Army Institute of Research, Silver Spring, Maryland

## Abstract

**Background:**

A safe and effective vaccine against dengue is needed to address an unmet medical need that affects a large portion of the world’s population. Takeda’s live attenuated tetravalent dengue vaccine candidate (TAK-003) has shown protection in an ongoing Phase 3 efficacy trial. TAK-003 contains an attenuated dengue type 2 virus (DENV-2), and 3 genetically modified viruses in which the structural proteins from each of the serotypes 1, 3 and 4 have been placed into the DENV-2 backbone. Exploratory immunological assessments have been a part of the TAK-003 clinical development plan to better understand the mechanisms of action of TAK-003, and to identify immune response signatures that may correlate with protection.

**Methods:**

Cellular and humoral immune responses elicited by vaccination in dengue-naïve and dengue-exposed individuals were measured across several clinical trials. For the humoral response, several methods were used to measure the magnitude and characteristics of the antibodies following vaccination with TAK-003 including studies of neutralizing antibodies, antibodies that bind to the viral components of the vaccine, the affinity and complement fixing capabilities of antibodies specific to structural proteins, and additionally the level of antibodies specific to nonstructural protein 1 (NS1).

**Results:**

A multifunctional cellular immune response was found following vaccination that primarily targeted nonstructural proteins in the DENV-2 backbone and was cross reactive to epitopes found in the other serotypes. The vaccine elicited neutralizing antibodies with high tetravalent seropositivity rates among participants. Further assessment of this response revealed that it consists of serotype-specific and cross-reactive neutralizing antibodies against all four serotypes. In addition, sera from vaccinated individuals neutralized genotypically diverse dengue strains. In addition to antibodies specific to structural components, antibodies to DENV-2 NS1 that were cross reactive to the NS1 proteins of the other serotypes were found.

**Conclusion:**

The breadth of the cellular and humoral immune responses elicited by TAK-003 in vaccine recipients across a wide age range living in different endemicities aligns with the response profile expected of a multivalent live vaccine.

**Disclosures:**

**Ralph Braun, PhD**, **Takeda Pharmaceuticals** (Employee) **Mayuri Sharma, PhD**, **Takeda Pharmaceuticals** (Employee) **Christina DeMaso, n/a**, **Takeda Pharmaceuticals** (Employee) **Allan Parker, n/a**, **Takeda Pharmaceuticals** (Employee) **David Dominguez, n/a**, **Takeda Pharmaceuticals** (Employee) **Heather Watkins, n/a**, **Takeda Pharmaceuticals** (Employee) **Hansi Dean, PhD**, **Takeda Pharmaceuticals** (Consultant) **Lovkesh Karwal, n/a**, **Takeda Pharmaceuticals** (Employee) **Eduardo Nascimento, n/a**, **Takeda Pharmaceuticals** (Employee) **Nicole Messere, n/a**, **Takeda Pharmaceuticals** (Employee) **Isamu Tsuji, n/a**, **Takeda Pharmaceuticals** (Employee) **Melissa Zahralban-Steele, n/a**, **Takeda Pharmaceuticals** (Employee) **Jeffrey R. Currier, PhD**, **Takeda Pharmaceuticals** (Scientific Research Study Investigator) **Heather Friberg-Robertson, PhD**, **Takeda Pharmaceuticals** (Scientific Research Study Investigator)

